# Critical thinking disposition of medical students in Anhui Province, China: a cross-sectional investigation

**DOI:** 10.1186/s12909-023-04646-x

**Published:** 2023-09-08

**Authors:** Jinxia Zhai, Haisheng Zhang

**Affiliations:** 1 Department of Occupational and Environmental Health, School of Public Health, Anhui Medical University, Meishan Rd 81, Hefei, 230032 China; 2 Marxism School of Anhui Medical University, Meishan Rd 81, Hefei, 230032 China

**Keywords:** Critical thinking, Critical thinking disposition, Critical thinking skills, California critical thinking dispositions inventory, Medical education

## Abstract

**Objective:**

To investigate the critical thinking disposition of medical undergraduates.

**Methods:**

This cross-sectional study was performed on 426 students from four majors, including preventive medicine, maternal and children’s health care medicine, health inspection and quarantine, and food quality and safety. The survey was completed in May 2019 using the California Critical Thinking Dispositions Inventory-Chinese version (CTDI-CV).

**Results:**

A total of 435 questionnaires were distributed and 426 valid questionnaires were collected, with an effective rate of 97.93%. The CTDI-CV overall average score was 262.02 ± 34.74 points indicating an ambivalent disposition in medical undergraduate students. Only one of the subscales (maturity in judgment) had mean scores of 43.35 ± 8.23 indicating the positive disposition of students. Among them, males scored 257.42 ± 35.06 lower than females’ 264.82 ± 34.32, the difference was statistically significant. The target scores of preventive medicine, maternal and children’s health medicine, health inspection and quarantine, and food quality and safety were 265.17 ± 30.10, 260.26 ± 37.05, 271.73 ± 33.55, and 252.11 ± 39.87, respectively. The difference was statistically significant. Among the three dimensions of seeking truth, open mind, and cognitive maturity, the scores of males were 38.26 ± 7.48, 38.78 ± 6.46 and 41.03 ± 8.69, which were lower than females’ 39.97 ± 7.11, 40.48 ± 6.48 and 44.91 ± 7.60, respectively. The scores of food quality and safety students were 37.23 ± 7.08, 36.61 ± 7.41 and 40.57 ± 8.60, respectively, which were lower than the preventive medicine (39.98 ± 7.07, 40.60 ± 5.96 and 44.44 ± 6.97, respectively).

**Conclusion:**

Most medical students were found to have an ambivalent disposition which meant they were not disposed toward critical thinking. These findings suggested that more effective teaching methods should be taken to facilitate critical thinking disposition and problem-solving ability.

## Introduction

Due to the multifaceted and complex decisions of prevention doctors, high-level cognitive abilities and professional techniques are required. There is increasing recognition that prevention doctors facilitate to prevention and control of the disease for humans, including infectious diseases and chronic non-communicable diseases. To achieve these optimal disease prevention and control outcomes, prevention doctors are required to provide evidence-based, effective protective, and population services. Prevention doctors need well-developed cognitive skills which need positive critical thinking abilities to make independent decisions. Despite the importance of critical thinking disposition, there is limited evidence focused on thinking processes in prevention medical students. Hence, we try to investigate the distribution of critical thinking disposition in medical undergraduates in this study.

In the education domain, there is disagreement about the term “critical thinking”. Predominant researchers defined “critical thinking” as a rational process involving ‘interpretation, analysis, evaluation, inference, explanation, induction, numeracy, deduce, and self-regulation’ [1]. Other researchers considered the term critical thinking as a more subjective process, with emotion and relationships [2]. The Association of American Colleges and Universities defined critical thinking as “a habit of mind characterized by the comprehensive exploration of issues, ideas, artifacts, and events before accepting or formulating an opinion or conclusion.” [3, 4]

The evidence has identified that there is a correlation between leadership and positive disease outcomes, including precise decisions and more health services, lower incidence, prevalence, and mortality of diseases, and higher healthy satisfaction. The level of cognition, leadership, and critical thinking abilities exert crucial roles in judgment and the ability to decision-making. Critical thinking skills can develop the ability for decision-making and problem-solving [3], which facilitates prevention medical students to improve their critical thinking abilities. Thus, the ability to think critically is necessary for prevention medical students because it will help them to develop the abilities of decision-making and problem-solving in their future careers.

The most commonly used measures to evaluate critical thinking abilities are standardized, commercially available tools such as the California Critical Thinking Skills Test (CCTST) [5], Critical Thinking Disposition Assessment (CTDA-R) [6], California Critical Thinking Disposition Inventory (CCTDI) [7], Health Sciences Reasoning Test (HSRT) [3], Watson-Glaser Critical Thinking Appraisal (WGCTA) [8], and critical thinking abilities self-evaluation scale [9]. These tools are often used to assess formal logic and general thinking skills. In recent decades, growing evidence demonstrated which assessment of critical thinking abilities in nursing and midwifery undergraduate [8, 10–13] students and pharmaceutical undergraduates [3]. However, there is seldom literature related to the critical thinking abilities of prevention medical undergraduate students. Due to the importance of critical thinking for preventive medical students both in an academic and field context, critical thinking is a crucial component of preventive doctors’ everyday problem-solving and decision-making processes, especially during the period of the epidemic situation of infectious diseases such as coronavirus disease 2019.

### Purpose and research question

The primary aim of this study was to better understand the characteristics that elicit students’ critical thinking abilities in the medical university, which provides a scientific basis for curriculum adjustment and modification. The role of educators is to teach students how to monitor their thinking and make themselves to better resolve problems with more thoughtful thinking. In this current study, we focused on preventive medicine, food quality and safety, and health inspection and quarantine since critical thinking assessments were already available for these disciplines. Specifically, we investigated the features of critical thinking abilities in undergraduate students.

While most critical thinking instruments in medical contexts have undergone some form of validation to investigate the features of students in nurse disciplines, to our knowledge none have explored the characteristics of preventive medical students’ critical thinking. This research provides new insight into the characteristics of undergraduate students’ critical thinking abilities, which can further be applied by educators to incorporate more effective critical thinking opportunities and contents in the classroom. These findings suggested the status of critical thinking abilities at the undergraduate level at Medical University, which provides new insight into the way of curriculum modification.

## Method

### Subjects and procedure

A cross-sectional survey of medical undergraduates from Anhui Province university was conducted in May 2019 using a convenience sampling method. The investigation inclusion criteria were: (1) agreed to participate in the investigation and no history of participation in similar studies of critical thinking tests, (2) at least six months of the study period in three subjects of medical university. The investigation exclusion criteria were: unwilling students. The study was approved by Anhui Medical University’s ethical committee (No.20170291). Students were informed of the aim of the research. Informed consent was obtained from all subjects and the questionnaire was completed anonymously in class. A total of 435 students from three academic years enrolled in a survey of critical thinking. Finally, 426 students consented to participate in the research study, which consisted of 265 females and 161 males. After the allocation, nine students were excluded because they could not complete the questionnaire.

### Data collection and California critical thinking skills test

Data were collected in May 2019 using the California Critical thinking disposition inventory Chinese version (CTDI-CV), which assesses the critical thinking disposition of undergraduate medical students. CTDI-CV, revised from the CCTDI, is a self-reported questionnaire with a six-point Likert-type scale [14], which consisted of seven subscales: truth-seeking, open-mindedness, analyticity, systematicity, confidence in reasoning, inquisitiveness, and maturity in judgment. This inventory has a total of 10 items in each subscale, the scores of each item are from “1” (completely agree) to “6” (completely disagree”). Thus, the target score of the inventory ranged from 70 to 420, for that a higher score means a better level of critical thinking disposition. The assessment criteria of positive disposition are above 40 scores in the subscale and above 280 scores in inventory, respectively. Among these, the critical thinking disposition is divided into four groups, including strong opposition (≤ 210 points), ambivalence (ranging from 211 to 279 points), positive (ranging from 280 to 349 points), and strong positive (≥ 350 points). The validity index (CVI) of CTDI–CV was 0.89 and the overall Cronbach’s alpha of CTDI–CV was 0.90 [14, 15].

The investigator approached students in their classrooms and explained the aims and features of the study. Furthermore, the investigator elucidates the feature and instructions of the CTDI-CV on how the test would be taken were explicated to the participants. In addition, participants were requested not to discuss the test with other students.

### Statistical analyses

Statistical analysis was performed for all quantitative data by using SPSS software version 23.0. Data were analyzed using descriptive (frequency, mean and standard deviation). Critical thinking abilities evaluation data were analyzed by independent sample *t*-test or one-way analysis of variance (ANOVA) with LSD post hoc test or Tamhane’s test. A level of statistical *P* < 0.05 was set for all the tests and considered to be statistically significant.

## Results

### Characteristics of the subjects

Four hundred and thirty-five students completed the critical thinking disposition assessment. Among these, 426 were valid samples. However, 9 were incomplete in the questionnaire. Exclusions were based on the fact of nine students did not fulfill responded to all test items. Table 1 showed that the female participants were 265 (62.2%), and all were regular (generic) students (100%). In terms of academic levels, 53 students (12.4%) were in the fifth year; 139 (32.6%) in the fourth year, and 234 (54.9%) were in the second year. In terms of academic majors, 176 students (41.3%) were prevention medicine, 164 (38.5%) were in maternal and children’s health medicine, 30 (7.0%) were in health inspection and quarantine, and 56 (13.1%) were in food quality and safety.

**Table 1:** The total score and dimension score of California Critical Thinking Disposition Inventory of medical students in Anhui Province, China (n = 426)

	n (%)	Overall scores of critical thinking	Truth-seeking	Open-mindedness	Analyticity	Systematicity	Confidence in reasoning	Inquisitiveness	Maturity in judgment
gender									
male	161 (37.8%)	257.42 ± 35.06*	38.26 ± 7.48*	38.78 ± 6.46*	34.78 ± 5.83	36.09 ± 6.10	33.73 ± 6.92	34.74 ± 6.29	41.03 ± 8.69*
female	265 (62.2%)	264.82 ± 34.32	39.97 ± 7.11	40.48 ± 6.48	34.55 ± 5.75	36.66 ± 6.20	33.93 ± 7.55	34.31 ± 6.56	44.91 ± 7.60
	t value P value	t=-2.139 P = 0.033	t=-2.365 P = 0.019	t=-2.619 P = 0.009	t = 0.395 P = 0.693	t=-0.931 P = 0.352	t=-0.267 P = 0.789	t = 0.672 P = 0.502	t=-4.842 P = 0.000
year of study(level)									
fifth year	53 (12.4%)	263.64 ± 35.47	40.24 ± 7.44	39.66 ± 6.75	35.68 ± 6.28	36.04 ± 6.28	34.45 ± 6.89	35.06 ± 6.46	42.51 ± 8.32
fourth year	139 (32.6%)	260.82 ± 33.06	38.78 ± 7.27	39.95 ± 5.81	34.33 ± 5.60	35.91 ± 6.34	33.93 ± 7.43	34.40 ± 6.32	43.52 ± 7.75
second year	234 (54.9%)	262.37 ± 35.67	39.44 ± 7.29	39.82 ± 6.52	34.59 ± 5.75	36.85 ± 6.02	33.68 ± 7.35	34.38 ± 6.55	43.62 ± 8.51
	F value P value	F = 0.152 P = 0.859	F = 0.834 P = 0.435	F = 0.041 P = 0.960	F = 1.068 P = 0.345	F = 1.140 P = 0.321	F = 0.254 P = 0.776	F = 0.250 P = 0.779	F = 0.397 P = 0.673
subjects									
preventive medicine	176 (41.3%)	265.17 ± 30.10	39.98 ± 7.07*	40.60 ± 5.96*	34.51 ± 5.49	36.89 ± 5.76	33.84 ± 7.78	34.95 ± 6.45	44.44 ± 6.97*
maternal and children’s health medicine	164 (38.5%)	260.26 ± 37.05	39.04 ± 7.53	39.80 ± 6.59*	34.53 ± 5.98	36.04 ± 6.43	33.40 ± 6.96	33.92 ± 6.53	43.53 ± 8.93*
health inspection and quarantine	30 (7.0%)	271.73 ± 33.55	40.96 ± 6.98*	41.73 ± 5.71*	36.53 ± 6.61	38.27 ± 5.45*	35.93 ± 6.51	35.80 ± 6.51	42.50 ± 9.41
food quantity and safety	56 (13.1%)	252.11 ± 39.87	37.23 ± 7.08	36.61 ± 7.41	34.38 ± 5.52	35.36 ± 6.74	34.12 ± 7.20	33.84 ± 6.19	40.57 ± 8.60
	F value P value	F = 2.965 P = 0.032	F = 2.641 P = 0.049	F = 6.454 P = 0.000	F = 1.169 P = 0.321	F = 1.974 P = 0.117	F = 1.044 P = 0.373	F = 1.334 P = 0.263	F = 3.327 P = 0.020

Note: Mean ± standard deviation; *, *P* < 0.05

### The distribution of critical thinking disposition in medical students

The distribution of critical thinking disposition in medical students is listed in Table 2. In terms of critical thinking disposition, 31 students (7.3%) were negative and 259 students (60.8%) were ambivalent. Meanwhile, 133 students (31.2%) obtained overall mean scores equal or above 280, which means positive disposition; whereas only three students (0.7%) scored 350 and above, which means strong positive disposition.

**Table 2:** The distribution of critical thinking disposition in medical students

CTDI-CV numerical score range	CTDI-CV qualitative category	N (%)
≤ 210 scores	Negative	31 (7.3)
211 ~ 279 scores	Inconsistent/Ambivalent	259 (60.8)
280 ~ 349 scores	Positive	133 (31.2)
≥ 350 scores	Strong Positive	3 (0.7)

The CTDI-CV scores are presented in Table 3. The CTDI-CV overall average score was 262.02 ± 34.74 indicating ambivalent disposition. Only one of the subscales (maturity in judgment) had mean scores above 40 indicating positive disposition of students in the subscales. The mean score was found in maturity in judgment (43.45). The lowest score was found on confidence in reasoning subscale (33.85). The second lowest mean score was found on the inquisitiveness (34.47) subscale followed by analyticity (34.64) and systematicity (36.44). These four subscales (analyticity, systematicity, confidence in reasoning, and inquisitiveness) mean scores were between 30 and 39 showing ambivalent inclination of students toward critical thinking. There are two of the subscales had mean scores of nearly 40 with truth-seeking (39.33) and open-mindedness (39.84), which need more samples to identify the dispositions.

**Table 3:** Distribution in Scores of California Critical Thinking Disposition Inventory of medical students in Anhui Province, China (n = 426)

Subscale	Mean	SD	Min	P5	P25	Median	P75	P95	Max
Overall	262.02	34.74	131.00	196.35	245.00	265.00	284.25	309.00	399.00
Truth-seeking	39.33	7.29	10.00	26.00	35.00	40.00	44.00	50.00	59.00
Open-mindedness	39.84	6.52	15.00	27.35	36.00	41.00	44.00	49.00	58.00
Analyticity	34.64	5.77	16.00	26.00	31.00	34.00	38.25	44.00	57.00
Systematicity	36.44	6.16	13.00	25.00	33.00	37.00	41.00	45.00	57.00
Confidence in reasoning	33.85	7.31	13.00	21.00	30.00	34.00	39.00	44.65	56.00
Inquisitiveness	34.47	6.45	16.00	23.00	30.00	35.00	39.00	44.65	57.00
Maturity in judgment	43.45	8.23	12.00	26.00	39.00	45.00	49.00	54.00	60.00

Note: “SD” means standard deviation, “Min” means minimum, and “Max” means maximum

### The total scores of CTDI-CV of subgroup in medical students

The CTDI-CV scores in students were 262.02 ± 34.74. Among these, the CTDI-CV target scores in male (257.42 ± 35.06) is significantly lower than that in female (264.82 ± 34.32) (*t*=-2.139, *P* = 0.033). In terms of academic levels, the CTDI-CV scores are 263.64 ± 35.47, 260.82 ± 33.06, and 262.37 ± 35.67 in the fifth year, fourth year, and second year, respectively, whilst there is no significant difference among academic levels (*F* = 0.152, *P* = 0.859). In terms of academic majors, the CTDI-CV scores are 265.17 ± 30.10, 260.26 ± 37.05, 271.73 ± 33.55, and 252.11 ± 39.87 in preventive medicine, maternal and children’s health medicine, health inspection and quarantine, and food quality and safety, respectively, whilst there is a significant difference among academic levels (*F* = 2.965, *P* = 0.032). After ANOVA with LSD analysis, the results demonstrated that the CTDI-CV scores in students of preventive medicine and health inspection are significantly higher than that in food quality and safety.

### Seven subscale scores of CTDI-CV in medical students

To explore the critical thinking disposition in medical students, we analyzed the seven subscale scores of CTDI-CV in medical students. The results were showed in Table 3.

We analyzed the subscale scores in truth-seeking of CTDI-CV. The truth-seeking subscale scores in male (38.26 ± 7.48) is significantly lower than that of female (39.97 ± 7.11) (*t* =-2.365, *P* = 0.019). In terms of academic levels, truth-seeking subscale scores are 40.24 ± 7.44, 38.78 ± 7.27, and 39.44 ± 7.29 in the fifth year, fourth year, and second year, respectively, whilst no difference among groups (*F* = 0.834, *P* = 0.435). In terms of academic majors, truth-seeking subscale scores are 39.98 ± 7.07, 39.04 ± 7.53, 40.96 ± 6.98, and 37.23 ± 7.08 in preventive medicine, maternal and children’s health medicine, health inspection and quarantine, and food quality and safety, respectively, whilst significant difference among groups (*F* = 2.641, *P* = 0.049). After ANOVA with LSD analysis, the results demonstrated that the truth-seeking subscale scores in students of preventive medicine and health inspection are significantly higher than that in food quality and safety.

We analyzed the subscale scores in open-mindedness of CTDI-CV. The open-mindedness subscale scores in male (38.78 ± 6.46) is significantly lower than that of female (40.48 ± 6.48) (*t*=-2.619, *P* = 0.009). In terms of academic levels, open-mindedness subscale scores are 39.66 ± 6.75, 39.95 ± 5.81, and 39.82 ± 6.52 in the fifth year, fourth year, and second year, respectively, whilst no difference among groups (*F* = 0.041, *P* = 0.960). In terms of academic majors, open-mindedness subscale scores are 40.60 ± 5.96, 39.80 ± 6.59, 41.73 ± 5.71, and 36.61 ± 7.41 in preventive medicine, maternal and children’s health medicine, health inspection and quarantine, and food quality and safety, respectively, whilst significant difference among groups (*F* = 6.454, *P* < 0.05). After ANOVA with LSD analysis, the results demonstrated that the open-mindedness subscale scores in students of food quality and safety are significantly lower than the other academic majors.

We analyzed the subscale scores in the analyticity of CTDI-CV. The analyticity subscale scores are no difference between males (34.78 ± 5.83) and females (34.55 ± 5.75) (*t* = 0.395, *P* = 0.693). In terms of academic levels, analyticity subscale scores are 35.68 ± 6.28, 34.33 ± 5.60, and 34.59 ± 5.75 in the fifth year, fourth year, and second year, respectively, whilst no difference among groups (*F* = 1.068, *P* = 0.345). In terms of academic majors, analyticity subscale scores are 34.51 ± 5.49, 34.53 ± 5.98, 36.53 ± 6.61, and 34.38 ± 5.52 in preventive medicine, maternal and children’s health medicine, health inspection and quarantine, and food quality and safety, respectively, whilst no difference among groups (*F* = 1.169, *P* = 0.321).

We analyzed the subscale scores in the systematicity of CTDI-CV. The systematicity subscale scores in males (36.09 ± 6.10) and females (36.66 ± 6.20) (*t* =-0.931, *P* = 0.352). In terms of systematicity levels, systematicity subscale scores are 36.04 ± 6.28, 35.91 ± 6.34, and 36.85 ± 6.02 in the fifth year, fourth year, and second year, respectively, whilst no difference among groups (*F* = 1.140, *P* = 0.321). In terms of academic majors, systematicity subscale scores are 36.89 ± 5.76, 36.04 ± 6.43, 38.27 ± 5.45, and 35.36 ± 6.74 in preventive medicine, maternal and children’s health medicine, health inspection and quarantine, and food quality and safety, respectively, whilst no difference among groups (*F* = 1.974, *P* = 0.117).

We analyzed the confidence in reasoning subscale scores of CTDI-CV. The confidence in reasoning subscale scores in males (33.73 ± 6.92) and females (33.93 ± 7.55) (*t* =-0.267, *P* = 0.789). In terms of systematicity levels, confidence in reasoning subscale scores are 34.45 ± 6.89, 33.93 ± 7.43, and 33.68 ± 7.35 in the fifth year, fourth year, and second year, respectively, whilst no difference among groups (*F* = 0.254, *P* = 0.776). In terms of academic majors, confidence in reasoning subscale scores are 33.84 ± 7.78, 33.40 ± 6.96, 35.93 ± 6.51, and 34.12 ± 7.20 in preventive medicine, maternal and children’s health medicine, health inspection and quarantine, and food quality and safety, respectively, whilst no difference among groups (*F* = 1.044, *P* = 0.373).

We analyzed the subscale scores in the inquisitiveness of CTDI-CV. The inquisitiveness subscale scores in males (34.74 ± 6.29) and females (34.31 ± 6.56) (*t* = 0.672, *P* = 0.502). In terms of systematicity levels, inquisitiveness subscale scores are 35.06 ± 6.46, 34.40 ± 6.32, and 34.38 ± 6.55 in the fifth year, fourth year, and second year, respectively, whilst no difference among groups (*F* = 0.250, *P* = 0.779). In terms of academic majors, inquisitiveness subscale scores are 34.95 ± 6.45, 33.92 ± 6.53, 35.80 ± 6.51, and 33.84 ± 6.19 in preventive medicine, maternal and children’s health medicine, health inspection and quarantine, and food quality and safety, respectively, whilst no difference among groups (*F* = 1.334, *P* = 0.263).

Finally, we analyzed the subscale scores in the maturity of judgment of CTDI-CV. The maturity of judgment subscale scores in males (41.03 ± 8.69) is significantly lower than that of females (44.91 ± 7.60) (*t* =-4.842, *P* < 0.05). In terms of systematicity levels, maturity of judgment subscale scores are 42.51 ± 8.32, 43.52 ± 7.75, and 43.62 ± 8.51 in the fifth year, fourth year, and second year, respectively, whilst no difference among groups (*F* = 0.397, *P* = 0.673). In terms of academic majors, scores of maturity of judgment subscale are 44.44 ± 6.97, 43.53 ± 8.93, 42.50 ± 9.41, and 40.57 ± 8.60 in preventive medicine, maternal and child health medicine, health inspection and quarantine, and food quality and safety, respectively, whilst significant difference among groups (*F* = 3.327, *P* = 0.020). After ANOVA with LSD analysis, the results demonstrated that the maturity of judgment subscale scores in students of preventive medicine is significantly higher than that in food quality and safety.

## Discussion

This study investigates the critical thinking disposition in medical undergraduate students. The results indicate an ambivalent range of critical thinking disposition in medical undergraduate students, including truth-seeking, open-mindedness, and maturity in judgment. However, the present findings resemble those of Zia [16] and Nguyen [13], who investigate the perception and disposition of critical thinking in medical undergraduate students, whereas different from the critical thinking disposition in German nurses [17] and Thailand dental students [18] with a positive attitude. This suggests that critical thinking disposition appear ambivalent to students similar to other countries and areas. As we know, one aim of education is to enable students to critically think. However, it is difficult to teach the students to develop their critical thinking disposition and skills. Due to the Asia traditional culture, Asian students showed lower critical thinking abiliiesy because it is not emphasized in their school education [18–20]. Despite this, the critical thinking disposition of dental students in Thailand showed a positive predisposition. One of the important reasons is high school students should take the entrance examination of the General Aptitude Test to apply for admission to dental school [18]. In addition, previous studies have demonstrated positive critical thinking abilities and skills in medical students in some countries [7], especially after some invention tests [3, 21]. These findings collectively suggest that traditional teaching methods could not improve the critical thinking disposition of medical undergraduate students in a medical university in China.

Alternatively, the results further confirmed that critical thinking disposition in male students is lower than that of female students, including truth-seeking, open-mindedness, and maturity of judgment. The critical thinking abilities of students are related to reading and writing but are not easily influenced by educational methods. Some studies such as that of Boso suggests a positive critical thinking disposition with 296 points in nursing students [7], and Zia shows an ambivalent critical thinking disposition [16]. In addition, previous studies have indicated that critical thinking significantly differs between learning styles and by student characteristics including academic year [17], nationality, previous experience, and intervention [3, 13, 19, 20]. However, in the present study, there is no difference in critical thinking disposition by student characteristics including academic year. Moreover, there is a significant difference in critical thinking disposition among academic majors, including truth-seeking, open-mindedness, and maturity in judgment. Consequently, future experimental studies may help better reveal the relationship between critical thinking disposition and educational methods style, which could elucidate its effect on the critical thinking abilities and skills of students using different teaching methods.

Moreover, in this current study, a high score on the maturity of judgment subscale suggested that students might own the ability to cope with complex situations, which might mean they have the ability of inductive reasoning [21]. Interestingly, the lowest two scores were the confidence in reasoning subscale and the inquisitiveness subscale, which are important indicators for inductive reasoning. Above all, it might be attributed to the traditional culture in China, which required the students is regard teachers as authorities and passive, traditional, and didactic teaching strategies [21, 22]. As a result, it caused a decrease in curiosity and self-confidence. Furthermore, the scores of truth-seeking subscale and open-mindedness subscale are nearly 40 points, which indicated the abilities of inference and evaluations might be target developing abilities in Chinese medical students in recent years. The results reflected that students would want to evaluate using new information and tolerance of divergent views. Additionally, the scores on analyticity subscales and systematicity subscales indicated an ambivalent inclination in medical students. For these, lower systematicity meant disorganized and disordered thinking [23]. Some studies showed that there are some factors that affect critical thinking development, including students’ background, culture [24], and teaching methods [10, 18, 25–27].

## Limitations of the study

There are some limitations in this current study that should be mentioned. First, a limited sample pool in this study, which is undergraduate students enrolled in preventive medicine, maternal and children’s health medicine, health inspection and quarantine, and food quality and safety, limits the generalization of conclusions. It needs to recruit more subjects representative sample of undergraduate students in future studies. Second, for some reason, we have not included the individual information in this investigation, such as family status, neighbors and partners, urban or rural origin, and family economic levels. Finally, cross-sectional was selected in this study to investigate the nature and characteristics of critical thinking disposition in undergraduate students in medical universities. Further longitudinal studies and intervention field tests should be used to explore the effective method to develop the critical thinking disposition and critical thinking skills in undergraduate students in medical universities.

## Conclusion

The finding in this present study indicated ambivalent critical thinking disposition in medical undergraduate students. Furthermore, critical thinking disposition in male undergraduates are lower than that of female undergraduates, such as truth-seeking, open-mindedness, and maturity of judgment. Moreover, the academic year might not be the predictor of critical thinking disposition, and this result is inconclusive with the result of other studies [17, 28, 29]. In addition, academic majors are related to critical thinking disposition, such as truth-seeking, open-mindedness, and maturity of judgment. These findings provide a preliminary account of how students critical thinking might be associated with academic majors, including academic course.

## Data Availability

All data generated and analyzed during this study are included in this published article.
